# Supplementary figures and images for: Anatomy and Comparative Transcriptome Reveal the Mechanism of Male Sterility in Salvia miltiorrhiza
Source: Int J Mol Sci. 2023 Jun 17;24(12):10259. doi: 10.3390/ijms241210259 (PMC10299234; doi:10.3390/ijms241210259)

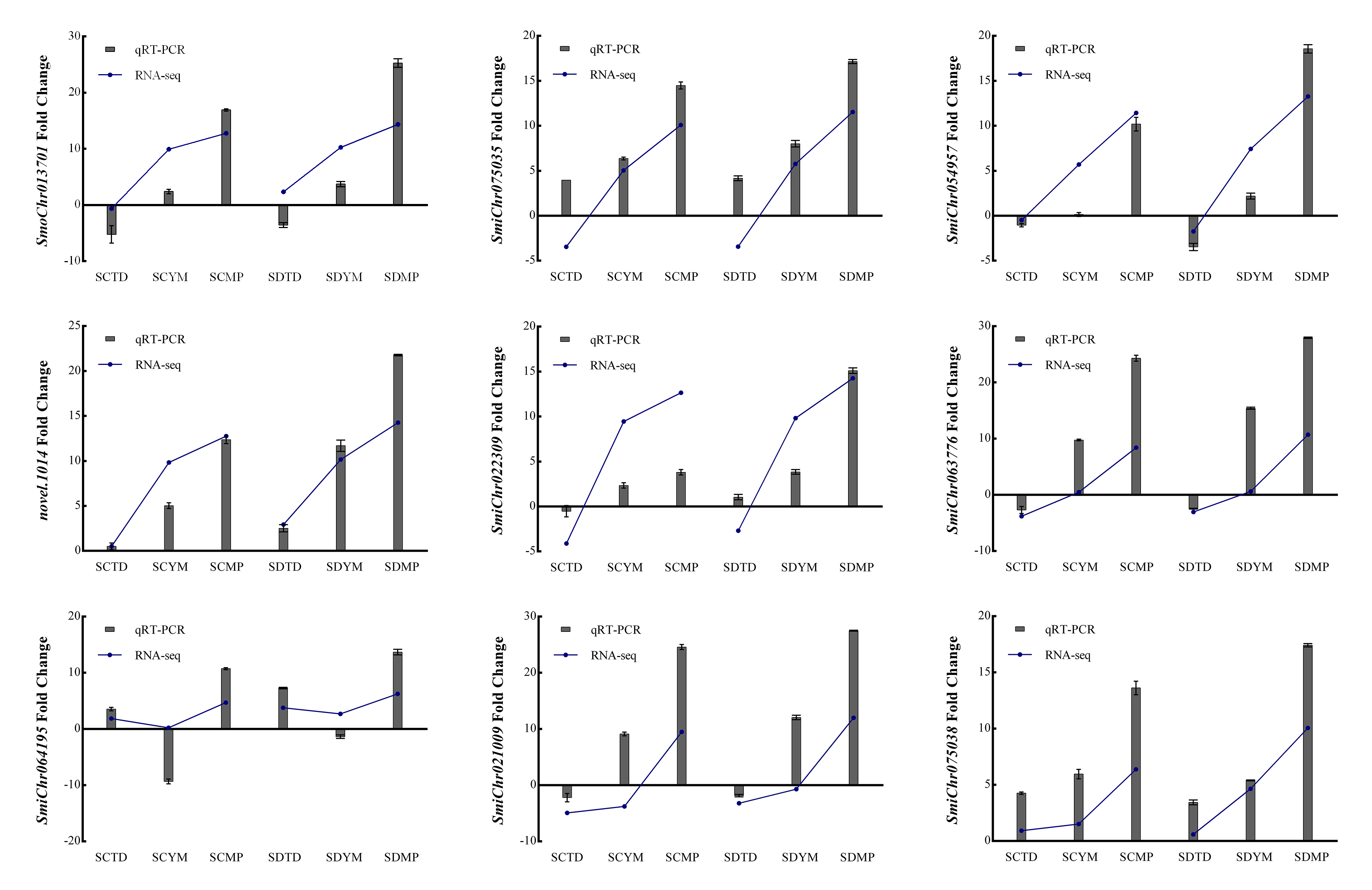

Supplement: Supplementary file 1 [file ijms-24-10259-s001.zip › Figure S1. Validation of DEGs by qRT-PCR.tif]

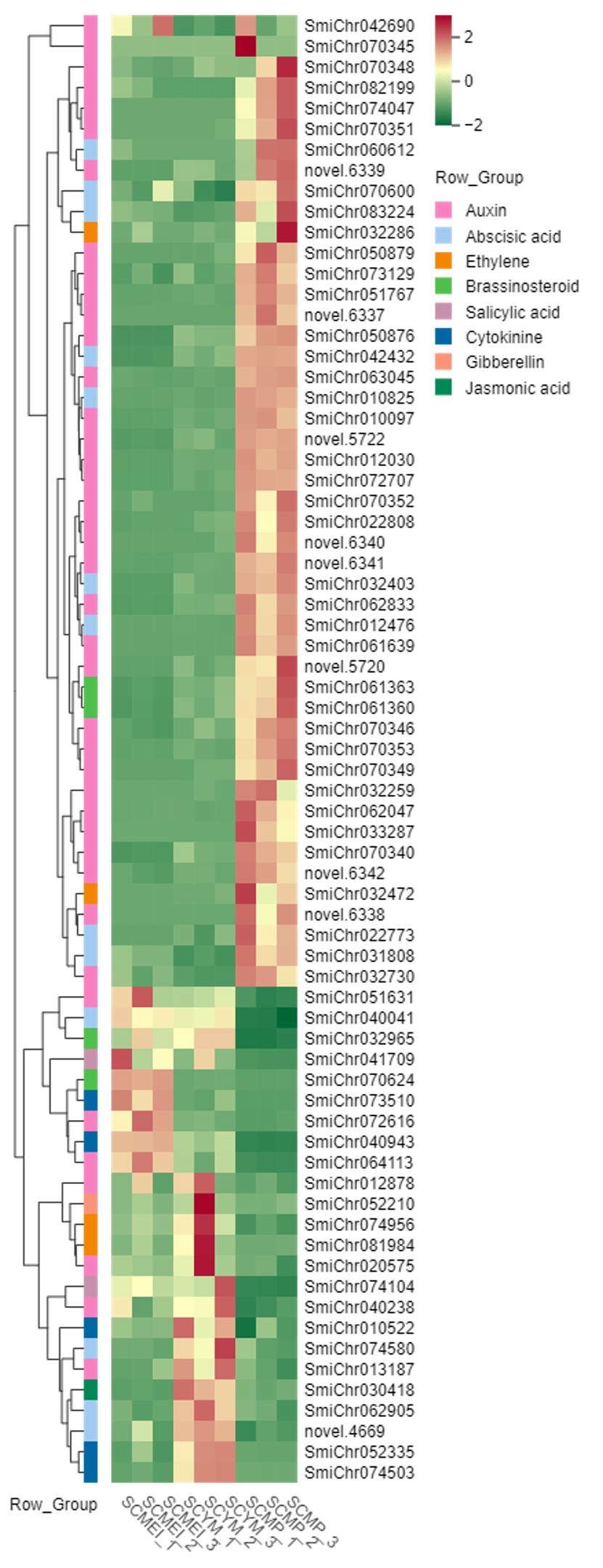

Supplement: Supplementary file 1 [file ijms-24-10259-s001.zip › Figure S2. DEGs of Plant hormone signal transduction in SC..png]

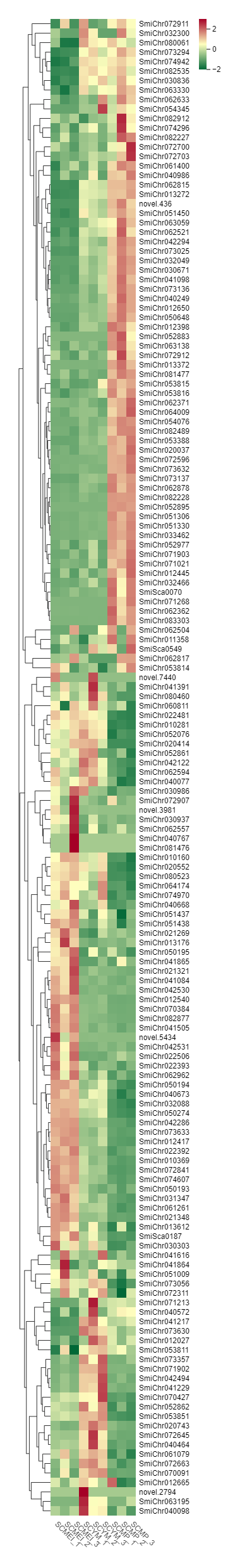

Supplement: Supplementary file 1 [file ijms-24-10259-s001.zip › Figure S3. DEGs of starch and sucrose metabolism in SC..png]
